# Ultrasound-based transient elastography combined with metabolic biomarkers enhances the diagnostic efficacy for pediatric MASLD

**DOI:** 10.1097/MD.0000000000050730

**Published:** 2026-09-18

**Authors:** Zefeng Bei, Yeying Xiao, Zhongbao Zuo, Jiahui He, Shanshan Wang, Ting Feng

**Affiliations:** a Department of Pediatrics, Hangzhou Xixi Hospital, Hangzhou, Zhejiang, China; b Department of Clinical Laboratory, Hangzhou Xixi Hospital, Hangzhou, Zhejiang, China; c Department of Ultrasound Medicine, Hangzhou Xixi Hospital, Hangzhou, Zhejiang, China.

**Keywords:** children, controlled attenuation parameter, metabolic biomarkers, nonalcoholic fatty liver disease, transient elastography

## Abstract

Nonalcoholic fatty liver disease, now encompassed within the updated metabolic dysfunction-associated steatotic liver disease (MASLD) nomenclature, is a common chronic liver disorder in children. This retrospective study evaluated the diagnostic performance of ultrasound-based transient elastography (TE) combined with metabolic biomarkers for clinically defined pediatric MASLD. A total of 334 children (278 in the MASLD group and 56 in the non-MASLD control group) were enrolled. MASLD status was determined retrospectively according to the Chinese Expert Consensus on the Diagnosis of Pediatric Nonalcoholic Fatty Liver Disease rather than liver biopsy. Liver stiffness measurement (LSM) and controlled attenuation parameter (CAP) were obtained using FibroTouch TE. Metabolic biomarkers covering liver function, lipid and glucose metabolism, inflammatory status, and uric acid metabolism were assessed. Receiver operating characteristic (ROC) analysis was used to evaluate discrimination, and exploratory combined models were constructed using logistic regression. Children with clinically defined MASLD showed significantly elevated LSM (7.52 ± 2.17 vs 5.76 ± 1.07 kPa, *P* < .001) and CAP (288.46 ± 46.76 vs 218.59 ± 28.16 dB/m, *P* < .001). CAP had the highest area under the ROC curve among single indicators (0.901, 95% confidence interval 0.868–0.935). The exploratory model incorporating CAP, LSM, C-reactive protein, uric acid, and homeostasis model assessment of insulin resistance yielded an apparent area under the ROC curve of 0.982 (95% confidence interval: 0.970–0.994), with sensitivity of 92.1% and specificity of 94.6% at the data-derived optimal cutoff. In this single-center retrospective cohort, combining TE parameters with metabolic biomarkers showed high apparent discrimination for clinically defined pediatric MASLD. Because the reference diagnosis was non-histologic and partly imaging-based, and because the model was evaluated in the same dataset in which it was developed without external validation, these estimates may be optimistic. Prospective multicenter validation against an independent reference standard is required before clinical implementation.

## 1. Introduction

Nonalcoholic fatty liver disease (NAFLD) has emerged as the most prevalent chronic liver disease in children and adolescents worldwide, with an estimated global prevalence of 2.57% in the general pediatric population and reaching up to 43% in obese children.^[1,2]^ NAFLD represents a spectrum of liver conditions ranging from simple steatosis to nonalcoholic steatohepatitis (NASH), which can progress to fibrosis, cirrhosis, and even hepatocellular carcinoma.^[3]^ The increasing prevalence of childhood obesity has made NAFLD a significant public health concern, emphasizing the urgent need for early and accurate diagnosis. In 2023, a multisociety Delphi consensus introduced the overarching term steatotic liver disease (SLD) and replaced NAFLD with metabolic dysfunction-associated steatotic liver disease (MASLD), which additionally requires at least 1 cardiometabolic risk factor.^[4]^

The gold standard for diagnosing MASLD remains liver biopsy. However, its invasive nature, potential complications, and sampling variability limit its widespread application in pediatric populations.^[4,5]^ Noninvasive diagnostic methods, including ultrasound, magnetic resonance imaging (MRI), and serum biomarkers, have been developed as alternatives. Among these, ultrasound-based transient elastography (TE) has gained considerable attention due to its noninvasive nature, reproducibility, and ability to simultaneously assess both liver steatosis and fibrosis.^[6,7]^

TE measures liver stiffness (LSM) and controlled attenuation parameter (CAP), which reflect liver fibrosis and steatosis, respectively. Previous studies have demonstrated the utility of TE in evaluating MASLD in adults.^[8,9]^ However, data on the application of TE in pediatric populations remain limited, particularly regarding optimal cutoff values applicable to diverse ethnic populations, and the optimal cutoff values for diagnosing MASLD in children have not been well established.^[10]^

Metabolic biomarkers, including liver function indicators, lipid metabolism parameters, glucose metabolism markers, and inflammatory indices, have been associated with MASLD pathogenesis.Insulin (INS) resistance, dyslipidemia, and chronic low-grade inflammation are key mechanisms underlying MASLD development.^[11,12]^ Recent studies have suggested that combining TE parameters with metabolic biomarkers may improve diagnostic accuracy for MASLD.^[13,14]^

This study aimed to evaluate the diagnostic efficacy of ultrasound-based TE combined with metabolic biomarkers for pediatric MASLD. Based on the multifactorial pathophysiology of MASLD, we hypothesized that the combination of CAP, LSM, and selected metabolic biomarkers would provide superior diagnostic performance compared to any single indicator alone. The findings of this study may contribute to the development of a noninvasive, accurate diagnostic approach for early detection of MASLD in children.

## 2. Materials and methods

### 2.1. Study population

This study was approved by the Ethics Committee of Hangzhou Xixi Hospital. This was a single-center, retrospective study conducted at Hangzhou Xixi Hospital. Children aged ≤18 years who visited the hospital from January 2020 to January 2026 were enrolled, including those diagnosed with MASLD (case group) and age- and sex-matched children without MASLD (control group). The inclusion criteria for the MASLD group were: Met the diagnostic criteria of the Chinese Expert Consensus on the Diagnosis of Pediatric Nonalcoholic Fatty Liver Disease (NAFLD)No history of alcohol consumption and No other causes of hepatic steatosis. The common exclusion criteria for both groups were as follows: Acute infection within the past 1 month; Preexisting chronic diseases, including chronic kidney disease, cardiovascular disease, autoimmune diseases, and other systemic disorders; Administration of drugs affecting liver function or lipid metabolism within the past 3 months; and Incomplete clinical data. Participants with incomplete clinical, laboratory, or TE data required for the present analyses were excluded during the screening process. Therefore, all participants included in the final analytic cohort had complete data for the variables analyzed in this study.”

This study was performed in accordance with the principles of the Declaration of Helsinki. Ethical approval was granted by the Ethics Committee of our hospital (2026 Research No. 041). Given the retrospective nature, anonymized processing of clinical data, and absence of invasive procedures or extra interventions, the requirement for informed consent was formally waived by the ethics committee. MASLD status was determined retrospectively according to the Chinese Expert Consensus on the Diagnosis of Pediatric, based on the available clinical manifestations, laboratory findings, exclusion of alternative causes of hepatic steatosis, and conventional imaging findings. Liver biopsy was not routinely performed in this pediatric cohort. Therefore, the reference classification used in the present study should be regarded as a consensus-based clinical diagnosis rather than a histological reference standard.

### 2.2. Examination and detection methods

#### 2.2.1. Ultrasound-based transient elastography (TE)

All participants underwent TE examination using a FibroTouch TE system (Model Pro5000, HiSKY, China) equipped with a variable-frequency probe (suitable for all populations). The examinations were performed by 2 experienced sonographers who were blinded to the participants’clinical information. Participants were placed in the supine position with the upper abdomen fully exposed; the right upper limb was elevated and abducted to expose the intercostal spaces. The probe was positioned at the 7th to 9th intercostal spaces over the right hepatic lobe (near the right anterior axillary line), held perpendicular to the skin with the measurement site fixed. A total of 10 valid measurements were obtained. The success rate was required to be ≥ 80%, and the interquartile range/median ratio was required to be ≤ 0.33. The median of the 10 valid measurements was taken as the final result. Liver stiffness measurement (LSM, unit: kPa) and (CAP, unit: dB/m) were recorded.

#### 2.2.2. Metabolic biomarkers detection

Venous blood samples (5 mL) were collected from all participants after an 8-hour overnight fast in the morning. The samples were centrifuged at 3000 rpm for 10 minutes to isolate serum, which was stored at −80°C until subsequent analysis.

Metabolic biomarkers included: Liver function indicators: Alanine aminotransferase (ALT), aspartate aminotransferase (AST), gamma-glutamyl transferase (GGT), and albumin (ALB); Lipid metabolism indicators: Total cholesterol (TC), triglyceride (TG), high-density lipoprotein cholesterol and low-density lipoprotein cholesterol,Glucose metabolism indicators: Fasting plasma glucose (FPG), INS, glycated hemoglobin (HbA1c), and homeostasis model assessment of INS resistance (HOMA-IR), calculated as (FPG [mmol/L] × INS [mIU/L])/22.5; Inflammatory indicators: White blood cell (WBC), neutrophil, lymphocyte (LYMPH), monocyte (MONO), platelet (PLT), C-reactive protein (CRP), and procalcitonin (PCT); and Uric acid (UA).

The following inflammatory ratios were calculated: NLR neutrophil-to-lymphocyte ratio (NLR) = NEUT count (10^9^/L)/LYMPH count (10^9^/L), MLR = monocyte-to-lymphocyte ratio (MLR) = MONO count (10^9^/L)/LYMPH count (10^9^/L), PLR platelet-to-lymphocyte ratio(PLR) = PLT count (10^9^/L)/LYMPH count (10^9^/L), and CAR = CRP (mg/L)/ALB (g/L).

Liver function and lipid metabolism indicators were measured using an automatic biochemical analyzer (Beckman Coulter AU5800). ALB was determined by the bromocresol green method. FPG was detected via the hexokinase method. INS was measured by chemiluminescence immunoassay using an Architect i2000 analyzer (Abbott). HbA1c was analyzed by high-performance liquid chromatography using an MQ-6000 hemoglobin analyzer. WBC, NEUT, LYMPH, MONO, and PLT counts were determined using an automatic hematology analyzer (Sysmex XN-9000 or Mindray BC-7500CS). CRP was detected by immunoturbidimetry using an ARISTO specific protein analyzer. PCT was measured by electrochemiluminescence immunoassay using a Roche cobas 601 analyzer. UA was measured by the uricase-peroxidase method.

### 2.3. Statistical analysis

Statistical analysis was performed using SPSS version 26.0 (IBM Corp., Armonk) and R version 4.2.1. Measurement data were expressed as mean± standard deviation (*x̅*±s) or median (interquartile range) (M[IQR]), and intergroup comparisons were performed using independent samples *t*-test or Mann–Whitney *U* test. Categorical data were expressed as frequency (percentage) (n[%]), and intergroup comparisons were performed using the *χ*^2^ test.

The correlations between TE parameters and metabolic biomarkers were analyzed using Pearson correlation analysis. Receiver operating characteristic (ROC) curve analysis was used to evaluate the diagnostic value of indicators. The area under the ROC curve (AUC) with a 95% confidence interval (CI) was calculated. The optimal cutoff value was determined based on the maximum Youden Index (sensitivity + specificity-1). Sensitivity, specificity, and Youden Index were calculated at the optimal cutoff point. The comparison of AUCs between different indicators was performed using the DeLong test.^[15]^ Combined models were constructed using multivariable logistic regression. Because model development and assessment were performed in the same retrospective cohort and no independent validation dataset was available, the resulting AUCs represent apparent (in-sample) discrimination rather than externally validated predictive performance. Accordingly, the combined models were considered exploratory. Data completeness was assessed before statistical analysis. Because participants with incomplete data required for the study analyses had been excluded during cohort selection, there were no missing values in the final analytic dataset. Therefore, no missing-data imputation was performed.

## 3. Results

### 3.1. Baseline characteristics of study population

A total of 432 children were initially screened, and after applying exclusion criteria, 334 children were included in the final analysis, comprising 278 in the MASLD group and 56 in the non-MASLD control group. The baseline characteristics of the 2 groups are presented in Table 1.

**Table 1:** Baseline characteristics of the 2 groups (n[%] or *x̅*±s).

Characteristic	Non-NAFLD group (*n* = 56)	NAFLD group (*n* = 278)	χ^2^/t	*P*-value
Gender			0.422	.516
Female	26 (46.43)	116 (41.73)		
Male	30 (53.57)	162 (58.27)		
Age (yr)	11.21 ± 2.78	11.25 ± 2.48	−0.094	.926
Height (cm)	149.14 ± 14.74	147.84 ± 14.23	0.608	.545
Weight (kg)	43.50 ± 6.82	46.91 ± 11.36	−2.167	.031
BMI (kg/m2)	19.61 ± 2.28	23.96 ± 3.64	−8.596	<.001

BMI = body mass index.

There were no significant differences in gender distribution (*χ*^2^ = 0.422, *P* = .516), height (*t* = −0.094, *P* = .926), and age (*t* = 0.608, *P* = .545) between the 2 groups. The weight (*t* = −2.167, P = .031) and body mass index of the MASLD group were significantly higher than that of the non-MASLD group (*t* = −8.596, *P* < .001), which was consistent with the close association between obesity and MASLD.

### 3.2. Intergroup comparison of ultrasound-based TE parameters

**Figure 1.**
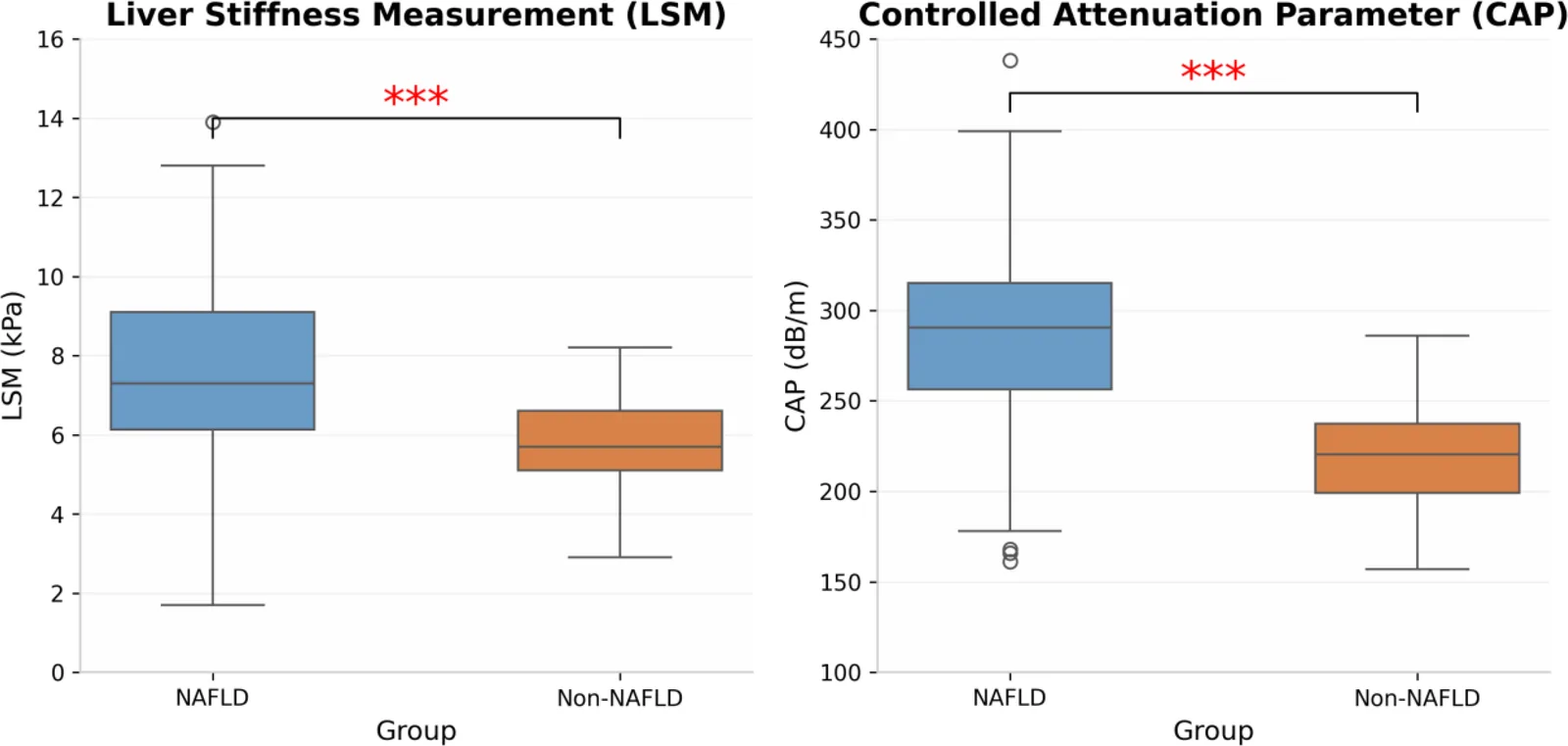
Intergroup comparison of transient elastography parameters. CAR = C-reactive protein-to-albumin ratio, LSM = liver stiffness measurement, NAFLD = nonalcoholic fatty liver disease.

As shown in Table 2 and Figure 1, the MASLD group had significantly higher LSM and CAP values than the non-MASLD group (all *P* < .05). These results indicate that children with MASLD have significant increases in both liver stiffness and fat attenuation compared to healthy children.

**Table 2:** Intergroup comparison of transient elastography parameters (*x̅*±s).

Parameter	Non-NAFLD group (*n* = 56)	NAFLD group (*n* = 278)	*t*	*P*-value
LSM (KPa)	5.76 ± 1.07	7.52 ± 2.17	−5.908	<.001
CAP (db/m)	218.59 ± 28.16	288.46 ± 46.76	−10.786	<.001

LSM = liver stiffness measurement. CAP = controlled attenuation parameter.

### 3.3. Intergroup comparison of metabolic biomarkers

**Figure 2.**
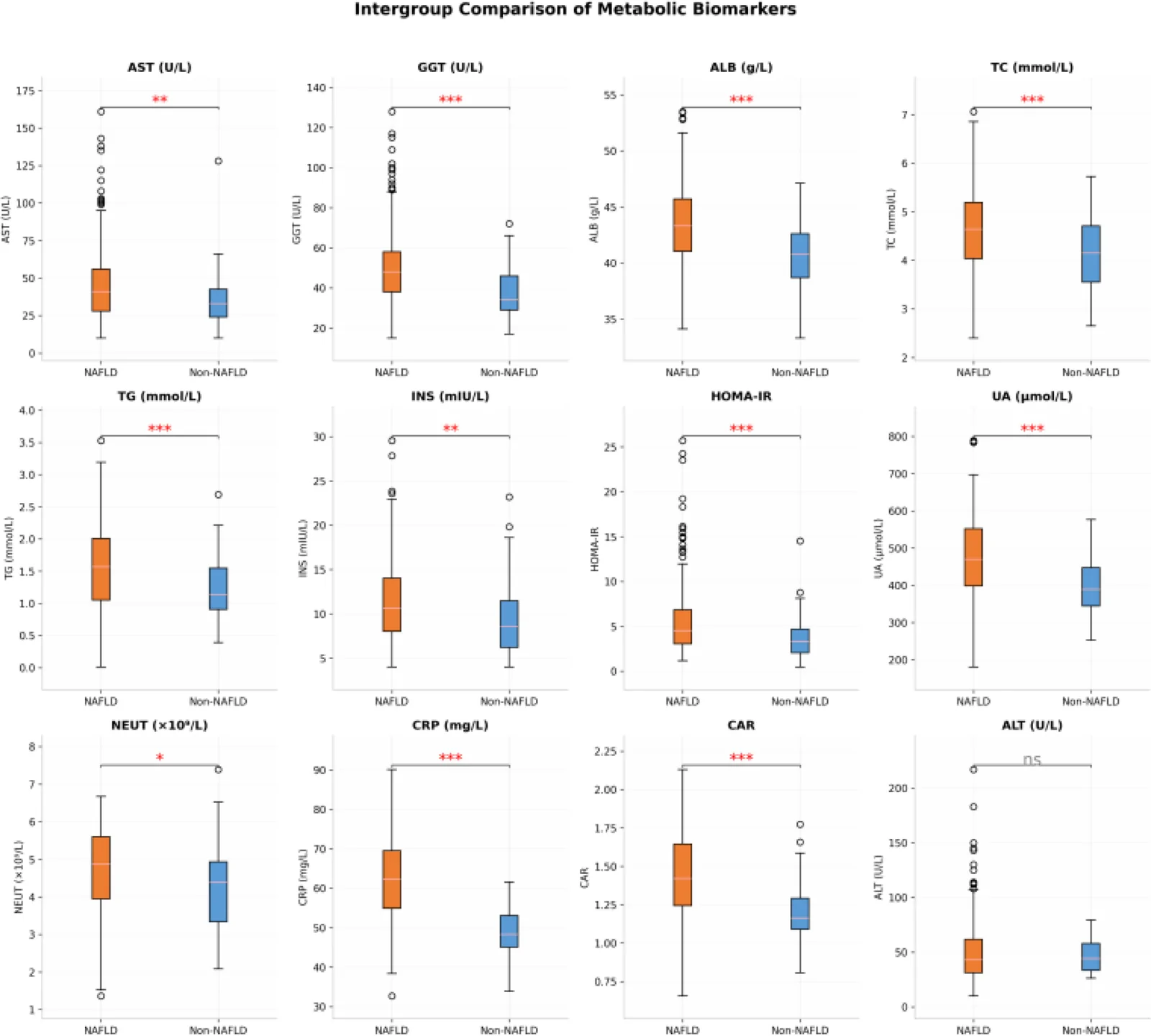
Intergroup comparison of metabolic biomarkers. ALB = albumin, AST = aspartate aminotransferase, CAR = C-reactive protein-to-albumin ratio, CRP = C-reactive protein, GGT = gamma-glutamyl transferase, HOMA-IR = homeostasis model assessment of insulin resistance, INS = insulin, LSM = liver stiffness measurement, NEUT = neutrophil, TC = total cholesterol, TG = triglyceride, UA = uric acid.

As shown in Table 3, the levels of AST, GGT, ALB, TC, TG, INS, HOMA-IR, UA, NEUT, CRP, and C-reactive protein-to-albumin ratio (CAR) in the MASLD group were significantly higher than in the non-MASLD group (all *P* < .05). However, no significant difference was found in the levels of ALT, HDL-C, LDL-C, FPG, HbA1c,WBC, LYMPH, MONO, PLT, PCT, NLR, MLR, and PLR (all *P* > .05) (Figure 2).

**Table 3:** Intergroup comparison of metabolic biomarkers (M [IQR], or *x̅*±s).

Indicator	Non-NAFLD group (*n* = 56)	NAFLD group (*n* = 278)	*Z/t*	*P-value*
Liver function indicators				
ALT (U/L)	42.50 (33.00, 55.50)	43.00 (31.00, 62.00)	−0.164	.870
AST (U/L)	33.00 (24.00, 43.00)	41.00 (28.00, 56.00)	−2.965	.003
GGT (U/L)	34.00 (29.00, 46.00)	48.00 (38.00, 58.00)	−5.106	<.001
ALB (g/L)	40.74 ± 3.30	43.40 ± 3.52	−5.438	<.001
Lipid Metabolism Indicators				
TC (mmol/L)	4.13 ± 0.74	4.61 ± 0.87	−4.304	<.001
TG (mmol/L)	1.23 ± 0.48	1.54 ± 0.70	−3.171	.002
HDL-C (mmol/L)	1.14 ± 0.17	1.10 ± 0.28	−1.214	.225
LDL-C (mmol/L)	2.28 ± 0.61	2.44 ± 0.68	−1.703	.092
Glucose Metabolism Indicators				
FPG (mmol/L)	4.91 ± 1.60	5.52 ± 2.56	−1.718	.087
INS (mIU/L)	8.58 (6.16, 11.48)	10.62 (8.02, 14.05)	−3.198	.001
HbA1c (%)	5.40 ± 0.33	5.45 ± 0.41	−0.854	.395
HOMA-IR	3.26 (2.03, 4.69)	4.54 (3.04, 6.85)	−4.347	<.001
Uric acid metabolism				
UA (μmol/L)	398.05 ± 76.68	474.48 ± 111.68	−6.244	<.001
Inflammatory Indicators				
WBC (×10^9^/L)	7.10 ± 2.05	7.55 ± 2.07	−1.500	.138
NEUT (×10^9^/L)	4.33 ± 1.16	4.72 ± 1.13	−2.339	.022
LYMPH (×10^9^/L)	3.85 ± 1.26	3.57 ± 0.99	−1.853	.065
MONO (×10^9^/L)	0.59 ± 0.25	0.63 ± 0.22	−1.214	.229
PLT (×10^9^/L)	244.57 ± 57.44	244.92 ± 53.71	−0.042	.966
CRP (mg/L)	48.55 ± 6.34	62.40 ± 10.50	−9.523	<.001
PCT (ng/mL)	0.19 ± 0.10	0.20 ± 0.14	−1.007	.316
NLR	1.28 ± 0.65	1.43 ± 0.55	−1.660	.101
MLR	0.18 ± 0.12	0.19 ± 0.08	−0.854	.394
PLR	71.41 ± 31.61	74.32 ± 27.37	−0.643	.522
CAR	1.20 ± 0.20	1.45 ± 0.27	−6.452	<.001

ALT = alanine aminotransferase, AST = aspartate aminotransferase, GGT = γ-glutamyl transpeptidase, ALB = albumin; TC = total cholesterol, TG = triglycerides, HDL-C = high-density lipoprotein cholesterol,LDL-C = low-density lipoprotein cholesterol, FPG = fasting plasma glucose, INS = fasting insulin, HbA1c = glycated haemoglobin, HOMA-IR = homeostasis model assessment of insulin resistance, UA = serum uric acid, WBC = white blood cell count, NEUT = neutrophil count,LYMPH = lymphocyte count, MONO = monocyte count, PLT = platelet count, CRP = C-reactive protein, PCT = procalcitonin, NLR = neutrophil-to-lymphocyte ratio, MLR = monocyte-to-lymphocyte ratio, PLR = platelet-to-lymphocyte ratio, CAR = C-reactive protein-to-albumin ratio.

### 3.4. Correlation analysis between transient elastography parameters and metabolic biomarkers

**Figure 3.**
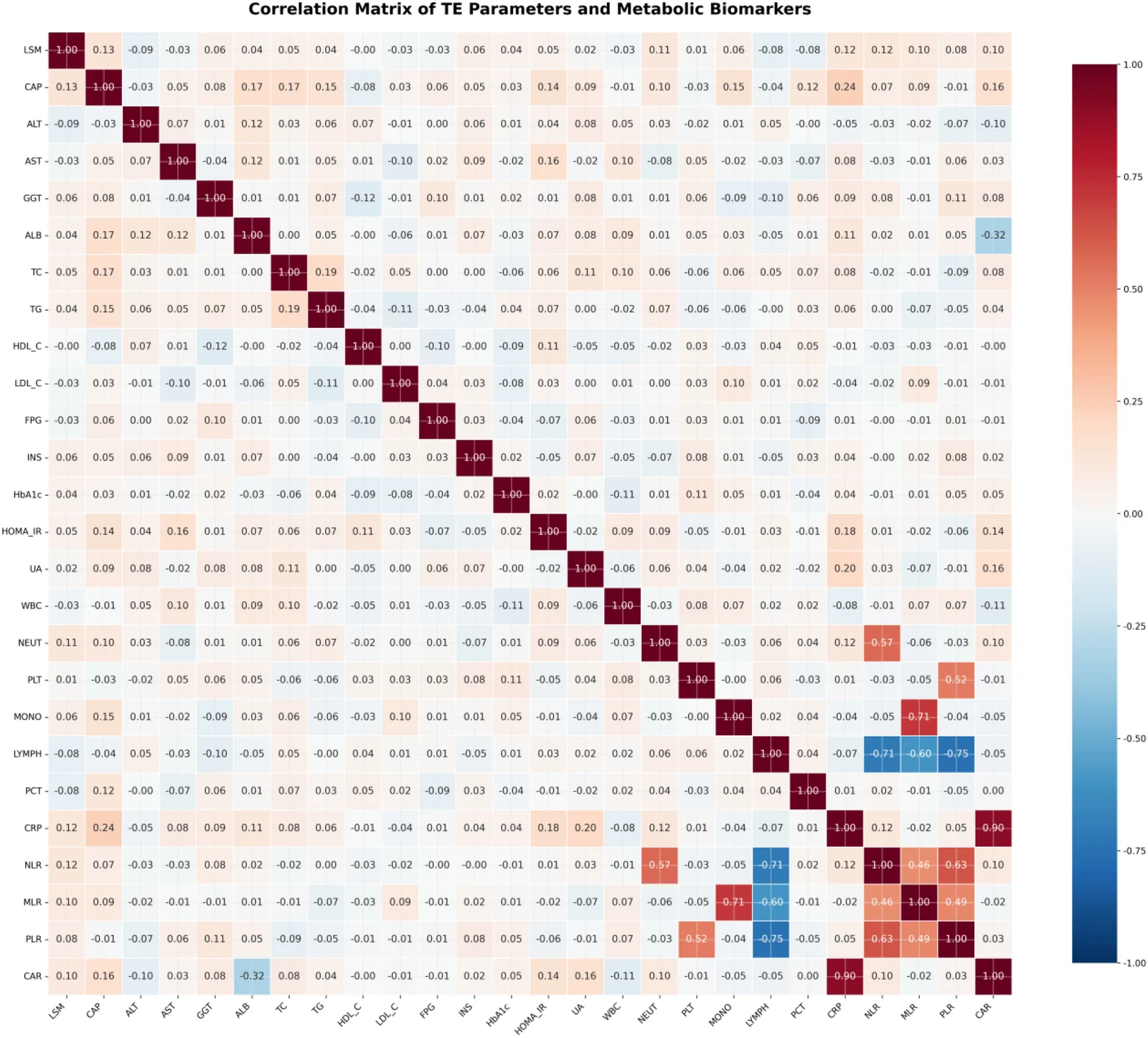
Correlation matrix of metabolic biomarkers. ALB = albumin, AST = aspartate aminotransferase, CAR = C-reactive protein-to-albumin ratio, CRP = C-reactive protein, GGT = gamma-glutamyl transferase, HOMA-IR = homeostasis model assessment of insulin resistance, INS = insulin, LSM = liver stiffness measurement, NEUT = neutrophil, TC = total cholesterol, TG = triglyceride, UA = uric acid.

Pearson correlation analysis revealed significant associations between TE parameters and metabolic biomarkers (Table 4 and Figure 3). For CAP, the strongest positive correlation was observed with CRP (*R* = 0.238, *P* < .001), followed by TC (*R* = 0.171, *P* = .002), ALB (*R* = 0.169, *P* = .002), CAR (*R* = 0.160, *P* = .003), MONO (*R* = 0.146, P = .007) HOMA-IR, (R = 0.144, P = .008), and TG (*R* = 0.150, *P* = .006). No significant negative correlations were found between CAP and any of the tested metabolic biomarkers (all *P* > .05). For LSM, significant positive correlations were identified with CRP (*R* = 0.125, *P* = .023), NEUT (*R* = 0.112, *P* = .040), and NLR (*R* = 0.124, *P* = .023). Other metabolic biomarkers (including ALT, AST, GGT, HDL-C, LDL-C, FPG, INS, HbA1c, UA, WBC, PLT, LYMPH, MLR, and PLR) showed no significant correlations with either CAP or LSM (all *P* > .05).

**Table 4:** Correlation coefficients between transient elastography parameters and metabolic biomarkers.

Metabolic Biomarker	CAP (dB/m)	*P*-value	LSM (kPa)	*P*-value
ALT	−0.022	.692	−0.089	.105
AST	0.047	.392	-0.031	.575
GGT	0.077	.162	0.059	.284
ALB	0.169	.002	0.042	.440
TC	0.171	.002	0.048	.385
TG	0.150	.006	0.040	.464
HDL_C	−0.080	.145	-0.003	.958
LDL_C	0.025	.645	−0.031	.578
FPG	0.064	.247	−0.026	.631
INS	0.048	.386	0.058	.287
HbA1c	0.028	.611	0.044	.426
HOMA_IR	0.144	.008	0.046	.399
UA	0.089	.106	0.015	.779
WBC	−0.007	.904	−0.032	.565
NEUT	0.102	.063	0.112	.040
PLT	−0.030	.590	0.011	.842
MONO	0.146	.007	0.058	.291
LYMPH	−0.044	.426	−0.080	.145
PCT	0.120	.028	−0.079	.151
CRP	0.238	.000	0.125	.023
NLR	0.073	.186	0.124	.023
MLR	0.094	0.085	0.096	.080
PLR	−0.011	.838	0.078	.155
CAR	0.160	.003	0.105	.056

ALB = albumin, AST = aspartate aminotransferase, CAR = C-reactive protein-to-albumin ratio, CRP = C-reactive protein, GGT = gamma-glutamyl transferase, HOMA-IR = homeostasis model assessment of insulin resistance, INS = insulin, LSM = liver stiffness measurement, NEUT = neutrophil, TC = total cholesterol, TG = triglyceride, UA = uric acid.

### 3.5. Diagnostic efficacy of individual and combined indicators

#### 3.5.1. Diagnostic efficacy of individual indicators

**Figure 4.**
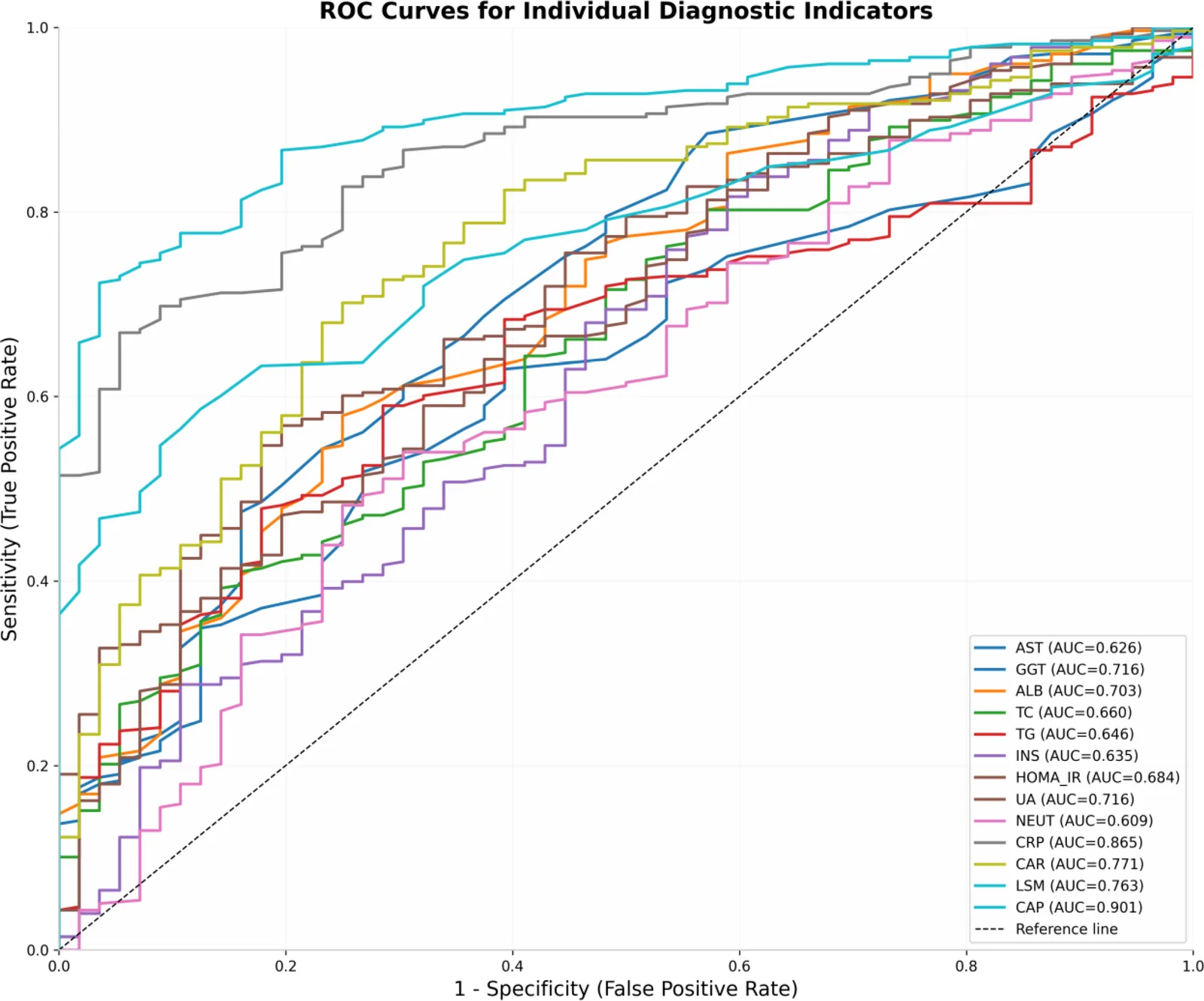
ROC curves for diagnostic indicators. ALB = albumin, AST = aspartate aminotransferase, CAR = C-reactive protein-to-albumin ratio, CRP = C-reactive protein, GGT = gamma-glutamyl transferase, HOMA-IR = homeostasis model assessment of insulin resistance, INS = insulin, LSM = liver stiffness measurement, NEUT = neutrophil, TC = total cholesterol, TG = triglyceride, UA = uric acid.

ROC curve analysis was performed to evaluate the diagnostic efficacy of TE parameters and metabolic biomarkers for childhood NAFLD with results summarized in Table 5 and Figure 4. Among all single indicators, the CAP exhibited the highest diagnostic performance, with an AUC of 0.901 (95% CI: 0.868–0.935, *P* < .001). At the optimal cutoff of 262.00 dB/m, it achieved a sensitivity of 72.3%, specificity of 96.4%, and Youden index of 0.687, indicating excellent diagnostic accuracy.

**Table 5:** Diagnostic efficacy of individual indicators for childhood NAFLD.

Indicator	AUC (95% *CI*)	Optimal cutoff	Sensitivity (%)	Specificity (%)	Youden index	*P*-value
CAP	0.901 (0.868–0.935)	262.00	72.3	96.4	0.687	<.001
CRP	0.865 (0.824–0.907)	57.80	66.9	94.6	0.615	<.001
CAR	0.771 (0.713–0.829)	1.29	70.1	75.0	0.451	<.001
LSM	0.763 (0.704–0.822)	6.90	58.6	87.5	0.461	<.001
GGT	0.716 (0.651–0.782)	50.00	47.5	83.9	0.314	<.001
UA	0.716 (0.651–0.781)	455.45	56.8	80.4	0.372	<.001
ALB	0.703 (0.636–0.770)	42.60	57.9	75.0	0.329	<.001
HOMA_IR	0.684 (0.615–0.753)	4.77	47.1	80.4	0.275	<.001
TC	0.660 (0.588–0.731)	4.82	41.0	83.9	0.249	<.001
TG	0.646 (0.573–0.719)	1.40	59.0	71.4	0.304	<.001
INS	0.635 (0.561–0.710)	7.35	83.8	39.3	0.231	<.001
AST	0.626 (0.550–0.701)	41.00	51.8	73.2	0.250	<.001
NEUT	0.609 (0.532–0.685)	4.73	54.0	69.6	0.236	<.001

ALB = albumin, AST = aspartate aminotransferase, CAR = C-reactive protein-to-albumin ratio, CRP = C-reactive protein, GGT = gamma-glutamyl transferase, HOMA-IR = homeostasis model assessment of insulin resistance, INS = insulin, LSM = liver stiffness measurement, NEUT = neutrophil, TC = total cholesterol, TG = triglyceride, UA = uric acid.

The diagnostic efficacy of other indicators in descending order of AUC was: CRP (AUC = 0.865, 95% CI: 0.824–0.907), CAR (AUC = 0.771, 95% CI: 0.713–0.829), LSM (AUC = 0.763, 95% CI: 0.704–0.822), GGT (AUC = 0.716, 95% CI: 0.651–0.782), UA (AUC = 0.716, 95% CI: 0.651–0.781), ALB (AUC = 0.703, 95% CI: 0.636–0.770), HOMA-IR (AUC = 0.684, 95% CI: 0.615–0.753), TC (AUC = 0.660, 95% CI: 0.588–0.731), TG (AUC = 0.646, 95% CI: 0.573–0.719), INS (AUC = 0.635, 95% CI: 0.561–0.710), AST (AUC = 0.626, 95% CI: 0.550–0.701), NEUT (AUC = 0.609, 95% CI: 0.532–0.685). All indicators showed statistically significant diagnostic value (all *P* < .001).

#### 3.5.2. Diagnostic efficacy of combined indicators

**Figure 5.**
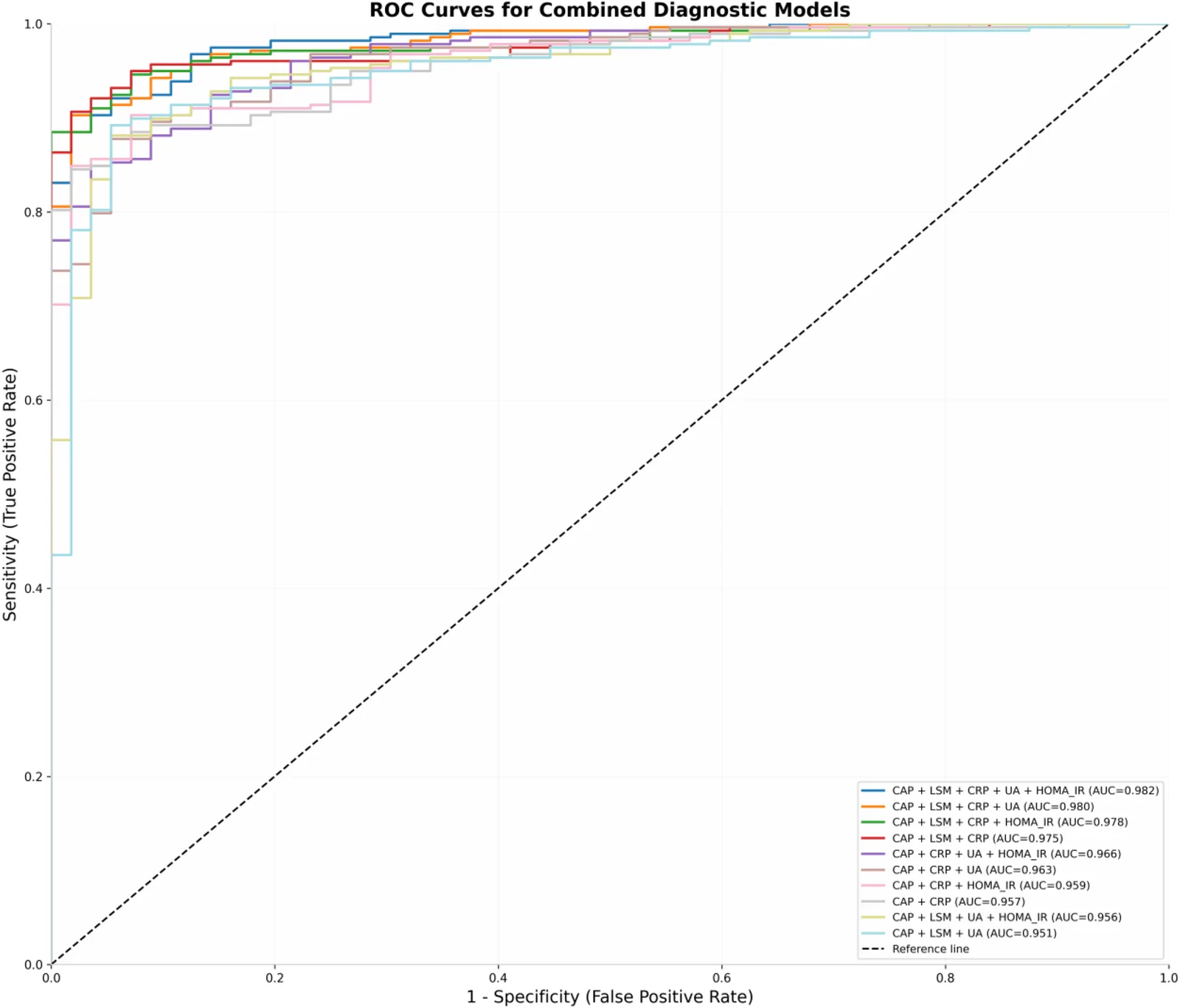
Diagnostic efficacy of combined indicators for childhood NAFLD. AUC = area under the ROC curve, CAR = C-reactive protein-to-albumin ratio, CRP = C-reactive protein, HOMA-IR = homeostasis model assessment of insulin resistance, LSM = liver stiffness measurement, NAFLD = nonalcoholic fatty liver disease. UA = uric acid.

To further improve diagnostic efficacy, we constructed combined diagnostic models using TE parameters and metabolic biomarkers based on AUC values. As shown in Table 6, the combined model of CAP + LSM + CRP + UA + HOMA-IR achieved the highest AUC of 0.982 (95% CI: 0.970–0.994), with a sensitivity of 92.1%, specificity of 94.6%, and a Youden Index of 0.867, which was significantly superior to CAP alone (*P* < .001) (Figure 5).

**Table 6:** Diagnostic efficacy of combined indicators for childhood NAFLD.

Combined model	AUC (95% *CI*)	Sensitivity (%)	Specificity (%)	Youden index	*P*-value of DeLong test (vs CAP)
CAP + LSM + CRP + UA + HOMA_IR	0.982 (0.970–0.994)	92.1	94.6	0.867	<.001
CAP + LSM + CRP + UA	0.980 (0.967–0.993)	90.3	98.2	0.885	<.001
CAP + LSM + CRP + HOMA_IR	0.978 (0.964–0.991)	88.5	100	0.885	<.001
CAP + LSM + CRP	0.975 (0.961–0.990)	90.6	98.2	0.889	<.001
CAP + CRP + UA + HOMA_IR	0.966 (0.949–0.984)	84.9	96.4	0.813	<.001
CAP + CRP + UA	0.963 (0.945–0.981)	87.8	94.6	0.824	<.001
CAP + CRP + HOMA_IR	0.959 (0.940–0.978)	90.3	92.9	0.831	<.001
CAP + CRP	0.957 (0.937–0.977)	88.1	94.6	0.828	<.001
CAP + LSM + UA + HOMA_IR	0.956 (0.935–0.976)	88.1	94.6	0.828	<.001
CAP + LSM + UA	0.951 (0.929–0.973)	89.2	94.6	0.839	<.001
CAP + LSM + HOMA_IR	0.945 (0.922–0.968)	90.6	91.1	0.817	<.001
S−CAP + LSM	0.940 (0.915–0.964)	89.9	92.9	0.828	<.001
CAP + UA + HOMA_IR	0.926 (0.898–0.954)	83.8	91.1	0.749	.039
CAP + UA	0.919 (0.890–0.949)	84.9	92.9	0.777	.092
CAP + HOMA_IR	0.911 (0.879–0.942)	80.9	91.1	0.72	.376
LSM + CRP	0.909 (0.877–0.941)	86	80.4	0.663	.778

CAR = C-reactive protein-to-albumin ratio, CRP = C-reactive protein, GGT = gamma-glutamyl transferase, HOMA-IR = homeostasis model assessment of insulin resistance, INS = insulin, LSM = liver stiffness measurement, NEUT = neutrophil, TC = total cholesterol, TG = triglyceride, UA = uric acid.

Other combined models also demonstrated improved diagnostic performance compared to single indicators. The CAP + LSM + CRP + UA model achieved an AUC of 0.980 (95% CI: 0.967–0.993), and the CAP + LSM + CRP + HOMA-IR model achieved an AUC of 0.978 (95% CI: 0.964–0.991). These findings indicate that combining TE parameters with metabolic biomarkers improved discrimination between children classified as having and not having MASLD within the present cohort. Given the consensus-based reference diagnosis and lack of independent validation, this performance should be considered apparent and exploratory.

## 4. Discussion

This study evaluated the diagnostic efficacy of ultrasound-based TE combined with metabolic biomarkers for pediatric MASLD. The results fully validated our initial hypothesis that the combination of CAP, LSM, and selected metabolic biomarkers yields significantly superior diagnostic performance for pediatric MASLD compared to any single indicator alone. The main findings were as follows: Children with MASLD exhibited significantly elevated LSM and CAP values compared to healthy controls, indicating increased hepatic fibrosis and fat accumulation; Multiple metabolic biomarkers, including AST, GGT, ALB, TC, TG, UA, HOMA-IR, and CAR, were significantly altered in the MASLD group; Among single indicators, CAP demonstrated the highest diagnostic efficacy with an AUC of 0.90 and most notably, the combined diagnostic model incorporating CAP, LSM, CRP, UA, and HOMA-IR achieved the highest AUC of 0.982, significantly improving diagnostic accuracy beyond any single parameter. Although the 5-variable model yielded a high AUC of 0.982, this result should be interpreted cautiously. The model was developed and evaluated within the same retrospective cohort, without an independent validation dataset. Consequently, the reported AUC reflects apparent discrimination and may be optimistically biased because of overfitting and cohort-specific characteristics. The relatively small and imbalanced control group may further increase the uncertainty surrounding model performance. Therefore, the present findings should be regarded as hypothesis-generating rather than evidence of established predictive accuracy. Appropriate internal validation and subsequent external validation in independent, preferably multicenter pediatric cohorts are required to determine model stability, generalizability, calibration, and clinical utility.

The prevalence of pediatric MASLD has increased dramatically worldwide, paralleling the rise in childhood obesity.^[15]^ Early detection is crucial for preventing disease progression to nonalcoholic steatohepatitis fibrosis, and cirrhosis. Liver biopsy, the gold standard for diagnosis, is invasive and impractical for widespread screening in children due to its procedural risks, sampling variability, and poor acceptability.^[16]^ Therefore, developing accurate, noninvasive diagnostic methods represents an urgent clinical priority. TE has emerged as a promising noninvasive tool for assessing liver steatosis and fibrosis simultaneously. In our study, CAP showed excellent diagnostic performance with an AUC of 0.901 and an optimal cutoff value of 262.00 dB/m, consistent with previous reports in both adult and pediatric populations. Notably, this cutoff value is highly consistent with 258.00 dB/m identified by Yang et al in a Chinese cohort of obese children,^[17]^ and 259 dB/m reported by Chaidez et al in an international pediatric MASLD study. This highlights the good applicability of our CAP cutoff value in Chinese pediatric populations and provides a reliable reference for the clinical diagnosis of MASLD in Chinese children. CAP measures ultrasound attenuation, which is directly related to hepatic fat content. It reflects the actual degree of hepatic steatosis and thus has high specificity and sensitivity for MASLD diagnosis.

Several studies have specifically investigated the diagnostic value of CAP and LSM in pediatric MASLD. Chaidez et al demonstrated that a CAP cutoff of ≥259 dB/m predicted hepatic steatosis grades 1 to 3 with an AUC of 0.98, sensitivity of 94%, and specificity of 91%.^[16]^ In a Chinese cohort of obese children, Yang et al identified an optimal CAP cutoff of 258.00 dB/m (AUC: 0.757) for MASLD diagnosis, while the optimal LSM cutoff was 4.65 kPa (AUC: 0.768).^[17]^ Shin et al reported that CAP achieved an AUC of 0.941 for diagnosing hepatic steatosis in Korean children with MASLD, with a cutoff value of 241 dB/m^18^. Additionally, Ferraioli et al established a CAP cutoff of 249 dB/m for detecting mild steatosis in overweight and obese Italian children.^[18]^ Regarding LSM, Nobili et al reported that FibroScan achieved an AUC of 0.98 for detecting mild fibrosis in children with MASLD, with a cutoff of ≥5.1 kPa.^[19]^ Similarly, Alkhouri et al demonstrated an AUC of 1.00 for diagnosing significant fibrosis using LSM with an S-probe in pediatric patients.^[20]^ These findings collectively support the clinical utility of CAP and LSM as reliable noninvasive biomarkers for evaluating hepatic steatosis and fibrosis in children with MASLD.

Our study extends these previous findings by systematically evaluating a comprehensive panel of metabolic biomarkers and identifying the optimal combination for diagnostic purposes. The combination of TE parameters with metabolic biomarkers significantly improved diagnostic accuracy, with the integrated model achieving an AUC of 0.982. This improvement aligns with the multifactorial pathophysiology of MASLD, which involves complex interactions among hepatic steatosis, INS resistance, dyslipidemia, and chronic inflammation. The inclusion of CRP in the combined model is particularly noteworthy. CRP reflects systemic inflammatory status, which is relevant to MASLD pathogenesis and prognosis.^[21]^ Similarly, elevated UA and HOMA-IR have been consistently associated with MASLD through mechanisms involving oxidative stress, mitochondrial dysfunction, and metabolic dysregulation.^[22,23]^ By integrating these metabolic indicators with TE parameters, our model captures the heterogeneity of MASLD pathophysiology more comprehensively than imaging alone. The superior performance of the combined model (AUC 0.982 vs 0.901 for CAP alone) suggests that a multi-parameter approach may be optimal for pediatric MASLD screening in clinical practice. This strategy mirrors the recent trend toward composite scoring systems in adult MASLD diagnosis, such as the FibroScan-AST (FAST) score and Agile scores, which integrate TE parameters with biochemical markers to enhance diagnostic accuracy in adult populations.

The strengths of this study include the relatively large sample size, the comprehensive evaluation of metabolic biomarkers, and the rigorous statistical analysis, including multiple imputation for missing data. However, several limitations should be acknowledged. First, the retrospective design may introduce selection bias. Second, an important limitation of this study is the absence of liver biopsy as the reference standard. MASLD was classified according to a consensus-based clinical diagnostic framework incorporating clinical, laboratory, exclusionary, and imaging information. Because CAP and LSM assess hepatic steatosis and stiffness and are conceptually related to imaging features contributing to the clinical diagnosis of MASLD, incorporation bias cannot be excluded. This overlap between the index tests and the reference classification may have inflated the apparent diagnostic performance of CAP, LSM, and the combined model. Accordingly, the reported AUCs should be interpreted as discrimination against the consensus-based clinical classification used in this retrospective cohort rather than diagnostic accuracy against biopsy-confirmed MASLD. Future prospective studies should validate these findings using an independent reference standard, preferably histopathology when clinically justified, or validated quantitative imaging methods such as MRI-PDFF for hepatic steatosis. Third, the study was conducted at a single center, which may limit generalizability. Fourth, the sample size of the control group (n = 56) is relatively small and imbalanced with the MASLD group (n = 278), which may reduce the test efficiency of some statistical analyses; future studies should adopt a study design with balanced sample sizes between groups to improve statistical power.

Future multicenter prospective studies with histological confirmation are needed to validate our findings. Furthermore, subsequent research should focus on the following aspects: first, further evaluating the positive predictive value (PPV), negative predictive value (NPV), and clinical application thresholds of this combined diagnostic model to provide more detailed reference for clinical screening and diagnosis; second, validating the applicability of the model in special pediatric populations such as normal-weight children and children of different age groups to expand its clinical application scope; third, exploring the value of the model in dynamic monitoring of MASLD disease progression and evaluation of therapeutic response, so as to provide a noninvasive tool for the whole-course management of pediatric MASLD.

Ultrasound-TE combined with metabolic biomarkers significantly enhances the diagnostic efficacy for pediatric MASLD. The integrated model incorporating CAP, LSM, CRP, UA, and HOMA-IR provides a robust and reliable noninvasive approach for the early detection of MASLD in children, with diagnostic performance superior to any single parameter alone. This model is of great clinical significance for the early screening and diagnosis of pediatric MASLD in clinical practice. However, because the reference diagnosis was not based on liver biopsy and potential incorporation bias cannot be excluded, the observed diagnostic performance should be considered preliminary. Prospective multicenter validation using an independent reference standard is required before this combined model can be recommended for routine clinical diagnosis.

## Author contributions

**Conceptualization:** Zefeng Bei, Yeying Xiao, Zhongbao Zuo, Jiahui He, Shanshan Wang, Ting Feng.

**Data curation:** Zefeng Bei, Yeying Xiao, Zhongbao Zuo, Jiahui He, Shanshan Wang, Ting Feng.

**Formal analysis:** Zefeng Bei, Yeying Xiao, Zhongbao Zuo, Jiahui He, Shanshan Wang, Ting Feng.

**Funding acquisition:** Ting Feng.

**Investigation:** Ting Feng.

**Writing – original draft:** Ting Feng.

**Writing – review & editing:** Ting Feng.
